# Sex-Related Differences in Outpatient Healthcare of Acute Coronary Syndrome: Evidence from an Italian Real-World Investigation

**DOI:** 10.3390/jcm12082972

**Published:** 2023-04-19

**Authors:** Raffaella Ronco, Federico Rea, Amelia Filippelli, Aldo Pietro Maggioni, Giovanni Corrao

**Affiliations:** 1 National Centre for Healthcare Research and Pharmacoepidemiology, University of Milano-Bicocca, 20126 Milan, Italy; raffaella.ronco@unimib.it (R.R.); afilippelli@unisa.it (A.F.); giovanni.corrao@unimib.it (G.C.); 2 Unit of Biostatistics, Epidemiology and Public Health, Department of Statistics and Quantitative Methods, University of Milano-Bicocca, 20126 Milan, Italy; 3 Department of Medicine, Surgery, and Dentistry, University of Salerno, 84081 Baronissi, Italy; 4 ANMCO Research Center, 50121 Florence, Italy; maggioni@heartcarefoundation.it

**Keywords:** acute coronary syndrome, sex-differences, healthcare, public health, real-world

## Abstract

At the time of first acute coronary syndrome (ACS) hospital admission, women are generally older and have more comorbidities than men, which may explain differences in their short-term prognosis. However, few studies have focused on differences in the out-of-hospital management of men and women. This study investigated (i) the risk of clinical outcomes, (ii) the use of out-of-hospital healthcare and (iii) the effects of clinical recommendations on outcomes in men vs. women. A total of 90,779 residents of the Lombardy Region (Italy) were hospitalized for ACS from 2011 to 2015. Exposure to prescribed drugs, diagnostic procedures, laboratory tests, and cardiac rehabilitation in the first year after ACS hospitalization were recorded. To evaluate whether sex can modify the relationship between clinical recommendations and outcomes, adjusted Cox models were separately fitted for men and women. Women were exposed to fewer treatments, required fewer outpatient services than men and had a lower risk of long-term clinical events. The stratified analysis showed an association between adherence to clinical recommendations and a lower risk of clinical outcomes in both sexes. Since improved adherence to clinical recommendations seems to be beneficial for both sexes, tight out-of-hospital healthcare control should be recommended to achieve favourable clinical benefits.

## 1. Introduction

Acute coronary syndrome (ACS) includes multiple manifestations of myocardial ischemia, including ST-segment elevation myocardial infarction (STEMI), non-ST-segment elevation myocardial infarction (NSTEMI) and unstable angina (UA). In Italy, twenty-eight-day case cardiac fatalities decreased by almost two-thirds during the 1990s [1], likely due to impressive improvements in medical treatments. This led to an increased number of patients who survived an ACS episode. Therefore, the clinical management of ACS patients after hospital discharge became a major challenge to improving long-term prognosis [2]. Although evidence-based guidelines have been developed for the secondary prevention of cardiovascular events and death [3,4], the risk of adverse outcomes in these patients is still high [5]. This is partially due to suboptimal adherence to current clinical practice guidelines [6,7].

Several studies have reported that women have worse short-term outcomes after ACS treatment. This is likely because they experience their first ACS episode at an older age when their clinical profile is already compromised by other comorbidities [8,9,10,11,12]. However, few studies have investigated differences in the out-of-hospital management of ACS between men and women, and those that have evaluated drug treatment and long-term prognosis observed conflicting findings [13,14,15].

Based on these premises, a large real-world study of a cohort of patients admitted to the hospital for their first episode of ACS was performed to evaluate sex-related differences in both out-of-hospital healthcare and short- and long-term clinical outcomes. A further aim was to assess whether sex modified the relationship between post-discharge healthcare and clinical outcomes.

## 2. Materials and Methods

### 2.1. Setting

Data used for this study were retrieved from the healthcare utilization databases of Lombardy, a region of Italy that accounts for about 16% of its population (about 10 million individuals). All Italian citizens have equal access to health care services as part of the National Health Service (NHS).

Automated healthcare utilization databases allow the Lombardy Region to collect various information, including (i) demographic and administrative data on NHS beneficiaries, (ii) private and public hospital discharge records coded according to the International Classification of Diseases, 9th Revision Clinical Modification (ICD-CM-9) classification system; (iii) outpatient drug prescriptions coded with the Anatomical Therapeutic Chemical (ATC) classification system; and (iv) data on outpatient services, including specialist visits and diagnostic examinations reimbursable by the NHS.

Records are linked between databases through a single identification code. To preserve privacy, each identification code is automatically converted into an anonymous code. Patient identification by the Regional Health Authority is only allowed upon request by judicial authorities.

Studies on the healthcare utilization databases of Lombardy in the field of cardiovascular diseases have been carried out [16,17,18]. Supplementary Table S1 lists the codes that were used to identify hospitalizations, prescriptions and information of interest to the current paper.

### 2.2. Cohort Selection

**Figure 1:**
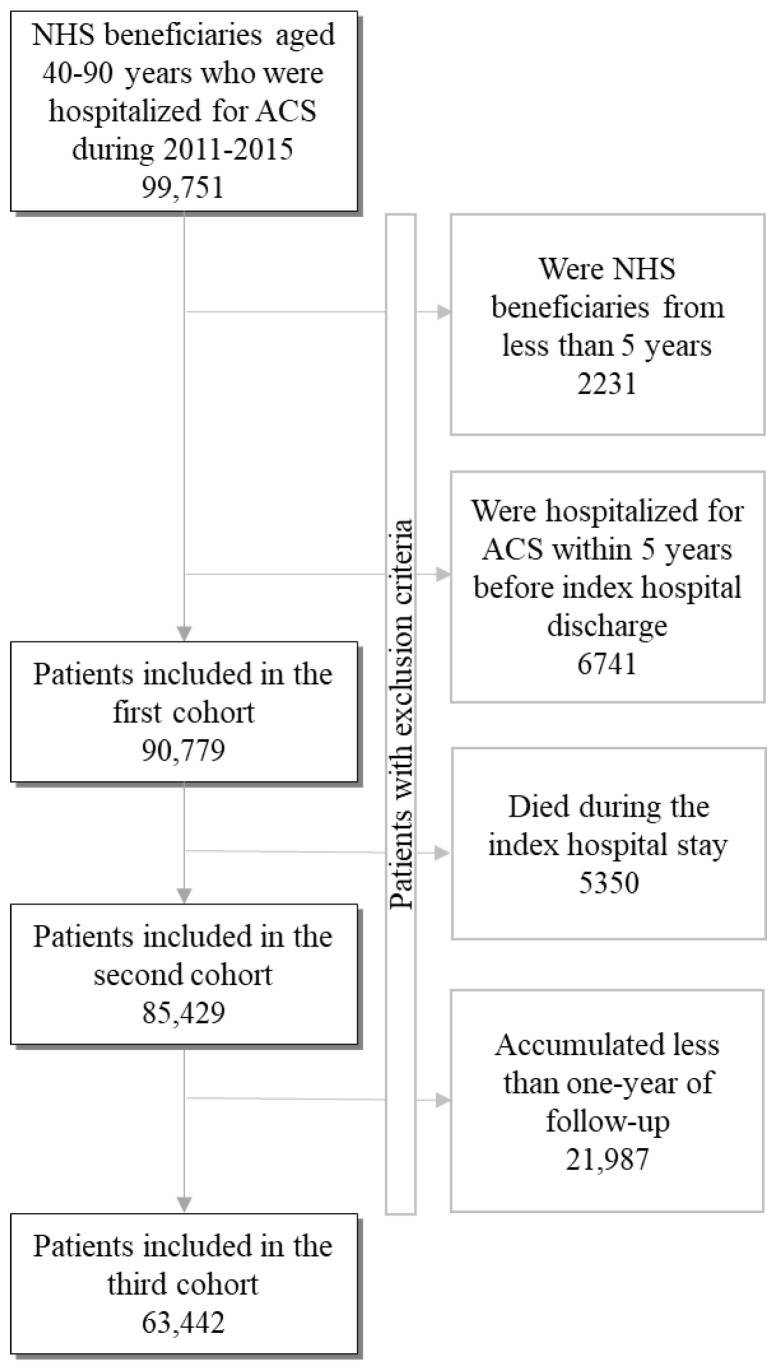
Flowchart of selection of the cohorts.

The target population consisted of residents of Lombardy aged 40–90 years. As shown in Figure 1, to assess (i) in-hospital all-cause mortality, (ii) out-of-hospital clinical outcomes, and (iii) healthcare provision to cohort members, three cohorts were identified and evaluated as described below.

First, patients who were hospitalized via the emergency room with an ACS diagnosis from 2011–2015 were identified, and the dates of admission and discharge from their first hospitalization during this period were recorded as “index admission” and “index discharge”, respectively. Patients who were beneficiaries of the NHS for less than five years prior to the index hospital admission, or who had a previous hospitalization for ACS during the same period, were excluded from the study cohort, as information from previous years was used to characterize cohort members. The remaining patients were included in the “first cohort” and were studied to evaluate in-hospital all-cause mortality.

Aiming to evaluate clinical outcomes after hospital discharge, subjects who survived the index hospitalization were selected for the “second cohort”. This latter group calculated person-years of follow-up from the index discharge until an end outcome (see below) or censoring event (death, emigration, or end of follow-up, i.e., 30 June 2018) occurred.

Finally, aiming to guarantee at least one year of observations to track outpatient services and treatments, patients who experienced a relevant clinical outcome (death or readmission for any cardiovascular causes) in the first year after hospital discharge were excluded from the “third cohort”. Patients of the third cohort were followed up until clinical outcomes or censoring as defined for the second cohort were met.

### 2.3. Cohort Baseline Characteristics

Baseline characteristics included those measured at the index admission, including sex, age, ACS type and comorbidities as drawn from in-hospital diagnoses and drugs dispensed within five years of the index admission: hypertension, dyslipidaemia, cerebrovascular disease, diabetes, chronic renal failure, chronic obstructive pulmonary disease (COPD), depression and cancer. Patient clinical status was assessed by the Multisource Comorbidity Score (MCS) [19], a comorbidity index that has been shown to predict mortality and other clinical outcomes in the Italian population better than other commonly used comorbidity scores. Four comorbidity profile categories were established: good (MCS: 0–4), intermediate (5–9), poor (10–14), and very poor (≥15).

### 2.4. Clinical Outcomes

In-hospital mortality of the first cohort was recorded. Of those who survived the index hospitalization and the first year of follow-up (i.e., patients included in the second and third cohorts), (i) hospital readmission for ACS, (ii) readmission for any cardiovascular cause, and (iii) all-cause mortality were evaluated as long-term clinical outcomes.

### 2.5. Adherence to Recommendations

To evaluate the use of out-of-hospital healthcare services in the first year following index hospital discharge, the following information was recorded: (i) prescribed drugs, (ii) access to outpatient clinical controls, and (iii) cardiac rehabilitation programs. Adherence to each of these healthcare categories was studied separately.

With respect to medications of interest, the prescription of renin–angiotensin system blockade agents (angiotensin-converting enzyme inhibitors or angiotensin receptor blockers), beta-blockers, statins and dual antiplatelet treatment (DAPT) was recorded. Two drug therapy outcomes were recorded: (i) starting drug treatment (i.e., at least one prescription) and (ii) adherence to drug treatment. To assess the latter, the period covered by the prescription was calculated according to the defined daily dose (DDD) metric. However, since beta-blockers are likely prescribed at doses lower than those established for treating hypertension following myocardial infarction [20], the corresponding dosage was carefully chosen by a working group of experts (Supplementary Table S2). For overlapping prescriptions, the individual was assumed to have completed the former one before starting the second. Adherence to drug therapy was assessed as the cumulative number of days during which the medication was available divided by the number of days of follow-up (365 days), a quantity defined as the “proportion of days covered” (PDC) [21]. Cohort members were considered adherent to each drug treatment if they had a PDC >75%, and to overall drug recommendations if they were adherent to at least 3 out of 4 therapies.

As far as outpatient services are concerned, cardiology visits, echo-electrocardiograms, and lipid profile tests were considered. Cohort members were classified as adherent to services if at least one outpatient service was prescribed during the first year after the ACS episode. Subjects were considered adherent to the overall recommendation if they underwent at least 2 of the 3 services.

Finally, participation in an outpatient cardiac rehabilitation program was recorded and patients were considered adherent if they used the program at least once.

### 2.6. Data Analysis

The chi-square test, or its version for the trend, was used to compare the demographic and clinical characteristics of men and women of the first cohort. As the sample size affects whether the results are significant, and about 100 thousand patients were included in our study (Figure 1), the standardized mean differences were also computed to better interpret the results [22]. A between-group mean standardized difference of <0.1 was considered negligible.

Aiming to evaluate associations between sex and intra-hospital mortality, logistic regression models were used to estimate the odds ratio (OR) and 95% confidence interval (CI). Regression was controlled for baseline age, MCS and comorbidities at the index date.

Since men and women had different clinical profiles and clinical characteristics, propensity score matching was used for the second and third cohorts. Propensity scores were derived through a logistic regression model that included age, type of ACS and comorbidities at baseline as covariates. Men and women were 1:1 matched using the nearest neighbour matching algorithm [23].

Of the matched and unmatched patients of the second cohort, the probability of experiencing a specific outcome (ACS, cardiovascular event or death) from the day after the index discharge until the end of follow-up was estimated using the cause-specific cumulative incidence function [24], which takes into account the competing nature of the considered outcomes (e.g., hospital readmission for ACS or other cardiovascular causes likely affects the subsequent probability of death). With this approach, a subject belonging to the second cohort was assumed to experience only one outcome (the one which comes first), and overall incidence at a given time was calculated as the sum of the individual cumulative incidence functions for each outcome.

In order to highlight significant differences in the out-of-hospital healthcare of the men and women in the third cohort, adherence to recommendations was compared using the McNemar test and the standardized mean differences.

To assess whether out-of-hospital healthcare has a different effect on clinical outcomes between men and women, a stratification approach was adopted [25]. Adjusted proportional hazard regression models were fitted to the men and women of the third cohort to estimate associations between adherence to clinical recommendations and the clinical composite outcome (i.e., hospital readmission for any cardiovascular cause or death). Heterogeneity between sex was tested by Cochran’s Q test [26].

All analyses were performed using Statistical Analysis System Software (version 9.4; SAS Institute, Cary, NC, USA). For all hypotheses tested, two-tailed *p*-values less than 0.05 were considered significant.

## 3. Results

### 3.1. Patients

Of the approximately 100 thousand NHS beneficiaries from the Lombardy region aged 40–90 years who were hospitalized for ACS from 2011 to 2015, 90,779 met the inclusion criteria for the first cohort, as shown in Figure 1.

There were 59,108 men (65%) and 31,671 women (35%), with mean ages of 67.6 and 75.1 years, respectively. STEMI was the most diagnosed type of ACS in both sexes (42% and 47% in women and men, respectively), followed by NSTEMI (36% and 33%, respectively) and unstable angina (21% and 20%, respectively). Women had more comorbidities (e.g., hypertension, COPD and depression) and a worse overall clinical profile based on the MCS (Table 1).

### 3.2. Clinical Outcomes

A total of 2492 (8%) women and 2843 (5%) men died during the index hospitalization. Compared with men, the unadjusted odds of death were 1.69 (95% CI, 1.60 to 1.79) times greater among women. In-hospital deaths were equivalent between sexes after adjusting for baseline characteristics (OR: 1.02, 0.96 to 1.08) (Table 2).

The 85,429 cohort members who survived the index hospitalization accumulated 273,228 person-years (86,189 in women and 187,039 in men) and generated 22,125 clinical outcomes (8780 in women and 13,345 in men). After matching, 20,079 couples were identified. The characteristics of cohort members included in the second cohort before and after matching are shown in Supplementary Table S3.

**Figure 2:**
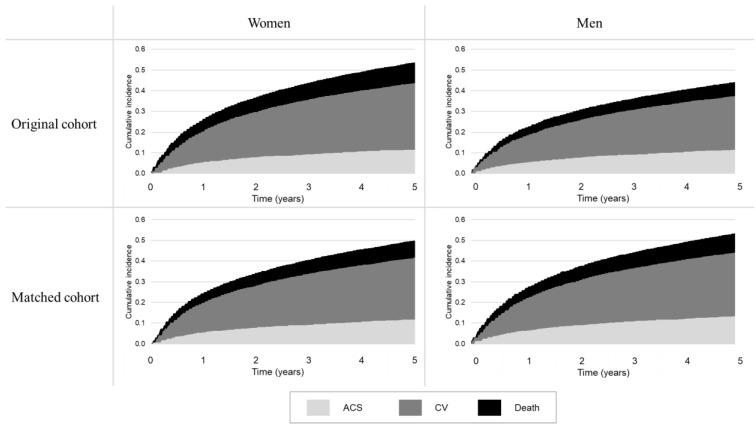
Cumulative incidences of health-related outcomes (ACS hospitalization, cardiovascular hospitalization, and all-cause mortality) among Propensity Score 1:1 matched and non-matched cohorts, according to sex.

Before matching, women had more clinical outcomes than men (54% vs. 44%, *p* < 0.001), especially in the first year after the hospital discharge (30% vs. 23%, *p* < 0.001) (Supplementary Figure S1). However, after matching, men had a slightly higher risk of clinical outcomes at one year (28% vs. 30%, *p* < 0.001) and five years of follow-up (50% vs. 53%, *p* < 0.001). The cumulative incidence functions are shown in Figure 2.

### 3.3. Out-Of-Hospital Healthcare

Of the 63,442 patients who did not experience a clinical outcome in the first year after hospital discharge (i.e., patients included in the third cohort), 20,450 were women and 42,992 were men. The matching procedure identified 14,354 couples. Characteristics of the original and matched third cohort are shown in Supplementary Table S4.

In the original cohort, women were less treated than men in all out-of-hospital healthcare services according to the McNemar test (Table 3). Higher differences were observed for some drug therapies (statins and DAPT), cardiology visits, and cardiac rehabilitation (standardized difference ≥ 0.1).

For example, 90% of men and 78% of women were prescribed statins, while the corresponding figures for DAPT were 74% vs. 60%, respectively. Among those prescribed drugs, men were more adherent to drug therapies (e.g., 77% vs. 65% among statin users). Only 5% and 2% of men and women went to cardiac rehabilitation.

Albeit the sex differences in all out-of-hospital healthcare services were confirmed in the matched cohort according to the McNemar test, some of these differences were reduced (e.g., cardiac controls) (Table 3).

### 3.4. Sex, Out-Of-Hospital Healthcare and Clinical Outcomes

The effects of out-of-hospital healthcare services on composite clinical outcomes are shown in Table 4.

Compared with non-adherent patients, those who adhered to drug therapies, outpatient services and cardiac rehabilitation programs had a lower risk of cardiovascular admission or death. There was no difference in the association between out-of-hospital healthcare services and clinical outcomes between men and women (all *p*-value > 0.05).

## 4. Discussion

The present study provides real-world evidence of sex-related differences in the health-related outcomes and out-of-hospital healthcare pathways of patients who were discharged after an episode of ACS.

At the time of their first ACS episode, women were older and had a worse clinical profile than men, findings confirmed by prior literature [27]. These characteristics may explain their higher risk of in-hospital mortality. Indeed, after adjusting for age and other baseline characteristics, there was no evidence that men and women had different in-hospital mortality risks. These results are consistent with those reported by the Italian National Outcome Program [28], showing the absence of sex differences in short-term mortality post-acute myocardial infarction. Among those who survived the first hospitalization, several patients experienced a second episode of ACS or another related cardiovascular event, mainly in the first year after discharge. This observation highlights the importance of a timely and efficient therapeutic and surveillance program that may reduce the risk of subsequent morbidity/mortality and promptly identify subsequent cardiovascular events.

The novel findings of the present study, however, rely on observed sex differences in the out-of-hospital provision of healthcare services. Women were less commonly prescribed recommended drug treatments than men, especially DAPT (74% of men vs. 60% of women). Lower DAPT prescription rates among women were also observed in previous studies [29], and Moriel et al. suggested that this could be explained by a greater prevalence of renal failure in women with STEMI [30]. While several patients were prescribed other drug therapies (about 80% for renin–angiotensin system blockers, statins and beta-blockers), only approximately three out of five patients adhered to treatment. As supported by several prior studies [29,30], women who were prescribed drug therapies were generally less willing to adhere to them than men. Weaker differences were observed among outpatient controls and diagnostic tests. To the best of our knowledge, no prior studies have compared access to these services between sexes.

Finally, cardiac rehabilitation was utilized by nearly double the number of men compared with women, although these programs were poorly attended by both sexes (less than 5% of the whole cohort). Since the use of outpatient cardiac rehabilitation programs has consistently been shown to be associated with more favourable cardiovascular outcomes, including mortality [31,32,33], there is still significant potential for cardiac rehabilitation to improve the long-term prognosis of ACS patients.

The most remarkable differences between men and women in receiving medical care are reported by studies in the United States, where services are paid for. Women usually have lower incomes than men and this may, at least in part, explain the large differences observed. However, in the Italian setting, this problem should not be so relevant, as women and men have an equal right to healthcare services.

As international guidelines do not include different out-of-hospital service recommendations between men and women [34], and there is no evidence that adherence to out-of-hospital healthcare improves long-term prognosis differently in men and women, emphasis should be placed on the greater use of healthcare resources by men compared with women.

The present study has several strengths. First, it was based on a very large and unselected population, made possible by the inclusion of nearly all citizens in Italy’s free healthcare system. Second, healthcare utilization databases provide highly accurate data, as all services claimed by the health providers for reimbursement by the Regional Health Authority are checked, and incorrect reports may have legal consequences. Finally, patients were identified based on their first hospitalization for ACS, allowing the complete sequence of post-discharge healthcare services supplied by the NHS to be identified.

Some limitations should be considered when properly interpreting our findings. Exposure misclassification may affect our findings in several ways. First, adherence to dispensed drugs was evaluated according to the DDD metric. Although guidelines do not recommend different drug therapy dosages for men and women, there may be differences in the prescribed dosage. Second, bias associated with our inability to account for out-of-pocket clinical evaluations, such as private cardiologist visits, should be noted [35,36]. However, estimates would not be biased if the use of out-of-pocket clinical evaluations similarly affected cohort members regardless of sex. Another limitation is that an intention-to-treat approach was adopted when considering healthcare exposure during follow-up, which was presumed to be consistent with the exposure level observed during the first year after index discharge, which may not be the case. Finally, like other administrative databases, the Lombardy database does not include clinical data (e.g., blood pressure), physical characteristics (e.g., body mass index) and lifestyle information (e.g., smoking status). In addition, the cause of death and other in-hospital data were not recorded in our database. Thus, we cannot examine the association between sex and intermediate clinical outcomes, and we cannot rule out the possibility that these unmeasured factors may confound associations between sex and clinical adherence. To minimize the potential for residual confounding, the propensity score matching design was adopted. Of course, this does not entirely avoid the problem of confounding, and thus further evidence is thus needed to confirm our findings.

## 5. Conclusions

Women were older and had a more compromised clinical profile at the time of their first ACS admission than men, which explains their observed short-term higher risk of death and cardiovascular events. Women were less treated and less adherent to clinical recommendations than men, although advantages derived from improved adherence to guideline-driven recommendations were expected for women just as much as for men. Tight out-of-hospital healthcare surveillance of ACS patients must be considered the cornerstone of optimizing the clinical outcomes of these patients.

## Figures and Tables

**Table 1:** Baseline characteristics of the 90,779 patients diagnosed with acute coronary syndrome in the Lombardy Region, Italy, 2011–2015.

	Whole Population (N = 90,779)	Women (N = 31,671)	Men (N = 59,108)	SD	*p*-Value *
Age (years)				0.66	<0.001
40–60	21,443 (23.6%)	3924 (12.4%)	17,519 (29.6%)		
61–70	20,798 (22.9%)	5368 (17.0%)	15,430 (26.1%)		
71–80	26,573 (29.3%)	10,063 (31.8%)	16,510 (27.9%)		
81–90	21,965 (24.2%)	12,316 (38.9%)	9649 (16.3%)		
ACS diagnosis				0.12	<0.001
STEMI	41,450 (45.7%)	13,366 (42.2%)	28,084 (47.5%)		
NSTEMI	30,812 (33.9%)	11,531 (36.4%)	19,281 (32.6%)		
Unstable angina	18,517 (20.4%)	6774 (21.4%)	11,743 (19.9%)		
Clinical profile ^†^				0.31	<0.001
Good	22,808 (25.1%)	5503 (17.4%)	17,305 (29.3%)		
Intermediate	28,062 (30.9%)	9666 (30.5%)	18,396 (31.1%)		
Poor	29,323 (32.3%)	12,337 (39.0%)	16,986 (28.7%)		
Very poor	10,586 (11.7%)	4105 (13.2%)	6421 (10.9%)		
Comorbidities ^‡^					
Hypertension	66,561 (73.3%)	25,997 (82.1%)	40,564 (68.6%)	0.32	<0.001
Dyslipidaemia	34,649 (38.2%)	12,227 (38.6%)	22,422 (37.9%)	0.01	0.047
Cerebrovascular disease	7148 (7.9%)	2987 (9.4%)	4161 (7.0%)	0.09	<0.001
Diabetes	21,373 (23.5%)	7856 (24.8%)	13,517 (22.9%)	0.05	<0.001
Chronic renal failure	1042 (1.2%)	334 (1.1%)	708 (1.2%)	0.01	0.054
COPD	28,421 (31.3%)	11,104 (35.1%)	17,317 (29.3%)	0.12	<0.001
Depression	17,305 (19.1%)	9215 (29.1%)	8090 (13.7%)	0.38	<0.001

ACS: acute coronary syndrome; NSTEMI: non-ST elevation myocardial infarction; SD: Standardized difference; STEMI: ST elevation myocardial infarction ^‡^ Comorbidity and Multisource Comorbidity Score both measured according to hospital admission and drug prescriptions experienced five years before the date of index admission. ^†^ Multisource Comorbidity Score is a comorbidity index obtained from inpatient diagnostic information and outpatient drug prescriptions, validated using Italian data. Four categories of clinical profiles were considered: good (MCS: 0–4), intermediate (5–9), poor (10–14) or very poor (≥15). * According to the chi-square test or its version for the trend.

**Table 2:** Odds ratio (OR) and 95% confidence intervals (CI) of the risk of intra-hospital mortality associated with sex. The first cohort was considered (see text).

	OR (95% CI)
Sex: Women vs. Men	1.02 (0.96–1.08)
Age (years)	
40–60	1.00 [Reference]
61–70	1.67 (1.44–1.92)
71–80	2.89 (2.53–3.30)
81–90	6.30 (5.23–7.19)
ACS diagnosis	
Unstable angina	1.00 [Reference]
NSTEMI	1.40 (1.28–1.53)
STEMI	2.16 (1.98–2.35)
Comorbidities ^‡^	
Hypertension	2.06 (1.85–2.30)
Dyslipidaemia	0.73 (0.69–0.78)
Cerebrovascular disease	1.28 (1.17–1.39)
Diabetes	1.15 (1.08–1.23)
Chronic renal failure	1.01 (0.81–1.25)
COPD	0.91 (0.86–0.97)
Depression	1.28 (1.20–1.36)
Clinical profile ^†^	
Good	1.00 [Reference]
Intermediate	1.09 (0.97–1.21)
Poor	1.54 (1.38–1.72)
Very poor	2.57 (2.26–2.92)

ACS: acute coronary syndrome. ^‡^ Comorbidity and Multisource Comorbidity Score both measured according to hospital admission and drug prescriptions experienced five years before the date of index admission. ^†^ Multisource Comorbidity Score is a comorbidity index obtained from inpatient diagnostic information and outpatient drug prescriptions, validated using Italian data. Four categories of clinical profiles were considered: good (MCS: 0–4), intermediate (5–9), poor (10–14) or very poor (≥15).

**Table 3:** Exposure to healthcare management strategies in the first year after discharge from the index hospital admission for acute coronary syndrome. The third cohort was considered (see text).

	Original Cohort (N = 63,442)				Matched Cohort (N = 28,708)			
	Women (N = 20,450)	Men (N = 42,992)	SD	*p*-Value *	Women (N = 14,354)	Men (N = 14,354)	SD	*p*-Value *
Drug therapies								
Prescription								
Renin–angiotensin system blockers	15,285 (74.7%)	33,716 (78.4%)	0.09	<0.001	10,726 (74.7%)	11,346 (79.0%)	0.10	<0.001
Beta-blockers	16,372 (80.1%)	35,820 (83.3%)	0.08	<0.001	11,665 (81.3%)	11,696 (81.5%)	0.01	<0.001
Statins	15,988 (78.2%)	38,621 (89.8%)	0.32	<0.001	11,471 (79.9%)	12,578 (87.6%)	0.21	<0.001
Dual antiplatelet treatment	12,290 (60.1%)	31,995 (74.4%)	0.31	<0.001	8772 (61.1%)	10,257 (71.5%)	0.22	<0.001
Three out of four	14,596 (71.4%)	35,810 (83.3%)	0.29	<0.001	10,442 (72.8%)	11,600 (80.8%)	0.19	<0.001
Adherence ^§^								
Renin–angiotensin system blockers	8772 (57.4%)	20,251 (60.1%)	0.05	<0.001	6165 (57.5%)	6829 (60.2%)	0.06	<0.001
Beta-blockers	8988 (54.9%)	21,005 (58.6%)	0.08	<0.001	6500 (55.7%)	6620 (56.6%)	0.02	<0.001
Statins	10,428 (65.2%)	29,711 (76.9%)	0.26	<0.001	7651 (66.7%)	9290 (73.9%)	0.16	<0.001
Dual antiplatelet treatment	6913 (56.3%)	20,527 (64.2%)	0.16	<0.001	5040 (57.5%)	6410 (62.5%)	0.10	<0.001
Three out of four	5966 (40.9%)	17,709 (49.5%)	0.17	<0.001	4396 (42.1%)	5507 (47.5%)	0.11	<0.001
Cardiac controls								
Cardiologic visits	14,461 (70.7%)	32,350 (75.3%)	0.10	<0.001	10,330 (72.0%)	10,597 (73.8%)	0.04	<0.001
ECO-Electrocardiograms	16,256 (79.5%)	35,668 (83.0%)	0.09	<0.001	11,588 (80.7%)	11,775 (82.0%)	0.03	<0.001
Test for lipid profile	16,524 (80.8%)	34,982 (81.4%)	0.01	<0.001	11,690 (81.4%)	11,708 (81.6%)	0.00	<0.001
Two out of three	16,230 (79.4%)	35,689 (83.0%)	0.09	<0.001	11,581 (80.7%)	11,779 (82.1%)	0.04	<0.001
Cardiac rehabilitation	413 (2.0%)	2092 (4.9%)	0.16	<0.001	327 (2.3%)	565 (3.9%)	0.10	<0.001

SD: Standardized difference. ^§^ Patients adherent to specific drug therapies among those who were prescribed at least one prescription of that treatment. * According to the McNemar test.

**Table 4:** Hazard ratios (HR), and 95% confidence intervals (CI), of the risk of composite outcome (cardiovascular hospitalization or death) associated with exposure to out-of-hospital healthcare, stratified by sex. The third cohort was considered (see text).

	Men	Women	*p*-Value *
Out-of-hospital healthcare ^†^			
Drug therapies	0.80 (0.75–0.85)	0.85 (0.79–0.92)	0.21
Cardiac controls	0.85 (0.79–0.91)	0.81 (0.76–0.87)	0.35
Cardiac rehabilitation	0.81 (0.67–0.97)	0.65 (0.48–0.87)	0.23

^†^ Patients were considered exposed to dispensed drugs if they adhered to at least 3 out of 4 drug therapies; patients were considered exposed to cardiac controls if they underwent at least 2 out of 3 services. * Test for heterogeneity was considered.

## Data Availability

The data that support the findings of this study are available from the Lombardy Region, but restrictions apply to the availability of these data, which were used under license for the current study, and so are not publicly available. Data are however available from the Lombardy Region upon reasonable request.

## Supplementary Materials

The following supporting information can be downloaded at: https://www.mdpi.com/article/10.3390/jcm12082972/s1, Table S1: Clinical diagnoses, drugs and outpatient services codes used for the study purpose; Table S2: Weights used to adjust the drug coverage of beta-blockers prescriptions; Table S3: Characteristics of cohort 2 members before and after matching; Table S4: Characteristics of cohort 3 members before and after matching.
