# Supplementary material for: Sex-Related Differences in Outpatient Healthcare of Acute Coronary Syndrome: Evidence from an Italian Real-World Investigation
Source: J Clin Med. 2023 Apr 19;12(8):2972. doi: 10.3390/jcm12082972 (PMC10140904; doi:10.3390/jcm12082972)
Supplement: Supplementary file 1 [file jcm-12-02972-s001.zip › jcm-2329888-supplementary.pdf]

Sex-Related Differences in Outpatient Healthcare of Acute Coronary Syndrome: Evidence from an Italian Real-World Investigation

Raffaella Ronco [1,2], Federico Rea [1,2,*], Amelia Filippelli [1,3], Aldo Pietro Maggioni [4] and Giovanni Corrao [1,2]

[1] National Centre for Healthcare Research and Pharmacoepidemiology, University of Milano-Bicocca, Milan 20126, Italy; raffaella.ronco@unimib.it (R.R.); afilippelli@unisa.it (A.F.); giovanni.corrao@unimib.it (G.C.)
[2] Unit of Biostatistics, Epidemiology and Public Health, Department of Statistics and Quantitative Methods, University of Milano-Bicocca, Milan 20126, Italy
[3] Department of Medicine, Surgery, and Dentistry, University of Salerno, Baronissi 84081, Italy
[4] ANMCO Research Center, Florence 50121, Italy; maggioni@heartcarefoundation.it
* Correspondence: federico.rea@unimib.it; Tel.: +39-026-448-5859

SUPPLEMENTARY MATERIAL

Table S1. Clinical diagnoses, drugs and outpatient services codes used for the study purpose.

	**Codes**
**Diseases †**	
Cardiovascular disease	390 – 459
Acute coronary syndrome	410.xx, 411.1x
Myocardial infraction	410.xx
Unstable angina	411.1x
Hypertension	401 – 405
Cerebrovascular disease	430.x – 438.x
Diabetes	250.x
Dyslipidaemia	272
Chronic kidney disease	585.x
COPD	491, 492, 494, 496
Cancer	140.x-208.x
Depression	296.2, 296.3, 296.82, 296.90, 298.0, 300.4, 309.0, 309.1, 311.x
**Rehabilitation hospitalization ‡**	56
**Drugs §**	
Antihypertensives	C02, C03, C07, C08, C09

Beta-blockers	C07
Renin-angiotensin system inhibitors	C09
Antiarrhythmics	C01B
Antidiabetics	A10
Statins	C10AA
Anticoagulant	B01AA, B01AE, B01AF
Antiplatelet	B01AC04, B01AC05, B01AC22, B01AC24
Drugs for pulmonary diseases	R03
Antidepressants	N06A
**Outpatient services**⫽	
Rehabilitation	MAC06, MAC07, MAC08
Electrocardiogram	89.50, 89.51, 89.52, 89.44.1, 89.48.2
Echocardiogram	88.72
Cardiological examination	89.01.3, 89.7A.3
Lipid profile	90.13, 90.14.3, 90.43.2

† According to the ICD-9-CM (International Classification of Disease, 9th Revision) system, ‡ According to the hospital ward coding; § According to the ATC (Anatomical-Therapeutic-Chemical) classification system;⫽According to the regional outpatients services coding.

Table S2. Weights used to adjust the drug coverage of beta-blockers prescriptions.

ATC	Weight
C07AA03	**2**
C07AA05	**1.3**
C07AA07	**1**
C07AB02	**3**
C07AB03	**1.5**
C07AB04	**1**
C07AB05	**1**
C07AB07	**4**
C07AB12	**2**
C07AG02	**6**
C07BB07	**4**
C07CB02	**1.5**
C07CB03	**1.5**

The drug coverage calculated according to the defined daily dose (DDD) was multiplied for the weights reported in this table, or, in an equivalent manner, the daily dose was calculated dividing the DDD for these weights.

Table S3. Characteristics of cohort 2 members before and after matching.

	Cohort 2 (N=85,429)				Matched cohort 2 (N=31,360)			
	**Women (N=29,173)**	**Men (N=56,256)**	**Standardized difference**	**p-value***	**Women (N=20,079)**	**Men (N=20,079)**	**Standardized difference**	**p-value***
**Age (years)**			0.66	<0.001			0.06	0.479
40 – 60	3,857 (13.2%)	17,288 (30.7%)			3,424 (17.1%)	3,187 (15.9%)		
61 – 70	5,210 (17.9%)	14,997 (26.7%)			4,166 (20.7%)	4,339 (21.6%)		
71 – 80	9,464 (32.4%)	15,566 (27.7%)			6,549 (32.6%)	7,062 (35.2%)		
81 – 90	10,642 (36.5%)	8,405 (14.9%)			5,940 (29.6%)	5,491 (27.3%)		
**ACS diagnosis**			0.12	<0.001			0.00	0.777
STEMI	12,051 (41.3%)	26,624 (47.3%)			8,501 (42.3%)	8,477 (42.2%)		
NSTEMI	10,680 (36.6%)	18,301 (32.5%)			7,145 (35.6%)	7,237 (36.0%)		
Unstable angina	6,442 (22.1%)	11,331 (20.1%)			4,433 (22.1%)	4,365 (21.7%)		
**Clinical profile**†			0.32	<0.001			0.11	<0.001
Good	5,248 (18.0%)	16,990 (30.2%)			3,920 (19.5%)	4,615 (23.0%)		
Intermediate	9,139 (31.3%)	17,840 (31.7%)			6,508 (32.4%)	6,028 (30.0%)		
Poor	11,207 (38.4%)	15,854 (28.2%)			7,334 (36.5%)	6,905 (34.4%)		
Very poor	3,579 (12.3%)	5,572 (9.9%)			2,317 (11.5%)	2,531 (12.6%)		
**Comorbidities**‡								
Hypertension	23,681 (81.2%)	37,971 (67.5%)	0.32	<0.001	15,664 (78.0%)	15,602 (77.7%)	0.01	0.456
Dyslipidaemia	11,280 (38.7%)	21,219 (37.7%)	0.02	0.007	7,888 (39.3%)	7,840 (39.1%)	0.00	0.624
Cerebrovascular disease	2,537 (8.7%)	3,671 (6.5%)	0.08	<0.001	1,658 (8.3%)	1,681 (8.4%)	0.00	0.678
Diabetes	7,062 (24.2%)	12,562 (22.3%)	0.04	<0.001	4,888 (24.3%)	4,768 (23.8%)	0.01	0.161

Chronic renal failure	308 (1.1%)	638 (1.1%)	0.01	0.300	204 (1.0%)	208 (1.0%)	0.00	0.843
COPD	10,216 (35.0%)	16,189 (28.8%)	0.13	<0.001	6,862 (34.2%)	6,865 (34.2%)	0.00	0.975
Depression	8,269 (28.3%)	7,391 (13.1%)	0.38	<0.001	4,467 (22.3%)	4,495 (22.4%)	0.00	0.737

†Multisource Comorbidity Score is a comorbidity index obtained from inpatient diagnostic information and outpatient drug prescriptions, and validated using Italian data. Patients were categorized as having good (MCS: 0-4), intermediate (5-9), poor (10-14) or very poor (≥ 15) clinical profile. ‡ Comorbidity and Multisource Comorbidity Score both measured according to hospital admission and drug prescriptions experienced five years before the date of index admission; * According to the chi-square test or its version for the trend.

Table S4. Characteristics of cohort 3 members before and after matching.

	**Cohort 3 (N=63,442)**				**Matched cohort 3 (N=28,708)**			
	**Women (N=20,450)**	**Men (N=42,992)**	**Standardized difference**	**p-value***	**Women (N=14,354)**	**Men (N=14,354)**	**Standardized difference**	**p-value***
**Age (years)**			0.64	<0.001			0.09	0.070
40 – 60	3,394 (16.6%)	15,265 (35.5%)			2,990 (20.8%)	2,810 (19.6%)		
61 – 70	4,248 (20.8%)	12,325 (28.7%)			3,387 (23.6%)	3,656 (25.5%)		
71 – 80	6,804 (33.3%)	10,879 (25.3%)			4,663 (32.5%)	4,985 (34.7%)		
81 – 90	6,004 (29.4%)	4,523 (10.5%)			3,314 (23.1%)	2,903 (20.2%)		
**ACS diagnosis**			0.14	<0.001			0.00	0.776
STEMI	8,529 (41.7%)	21,172 (49.2%)			6,278 (43.7%)	6,296 (43.9%)		
NSTEMI	7,177 (35.1%)	13,281 (30.9%)			4,826 (33.6%)	4,828 (33.6%)		
Unstable angina	4,744 (23.2%)	8,539 (19.9%)			3,250 (22.6%)	3,230 (22.5%)		
**Clinical profile**†			0.35	<0.001			0.11	0.005
Good	4,348 (21.2%)	14,942 (34.8%)			3,374 (23.5%)	3,868 (27.0%)		
Intermediate	7,240 (35.4%)	14,711 (34.2%)			5,265 (36.7%)	4,868 (33.9%)		
Poor	7,216 (35.3%)	10,701 (24.9%)			4,698 (32.7%)	4,447 (31.0%)		

Very poor	1,646 (8.1%)	2,638 (6.1%)			1,017 (7.1%)	1,171 (8.2%)		
**Comorbidities‡**								
Hypertension	15,843 (77.5%)	27,160 (63.2%)	0.32	<0.001	10,603 (73.9%)	10,569 (73.6%)	0.01	0.648
Dyslipidaemia	7,475 (36.6%)	14,997 (34.9%)	0.03	<0.001	5,287 (36.8%)	5,252 (36.6%)	0.01	0.668
Cerebrovascular disease	1,336 (6.5%)	2,000 (4.7%)	0.08	<0.001	858 (6.0%)	883 (6.2%)	0.01	0.536
Diabetes	4,106 (20.1%)	8,192 (19.1%)	0.03	0.002	2,854 (19.9%)	2,863 (20.0%)	0.00	0.894
Chronic renal failure	145 (0.7%)	334 (0.8%)	0.01	0.377	102 (0.7%)	104 (0.7%)	0.00	0.889
COPD	6,872 (33.6%)	11,150 (25.9%)	0.17	<0.001	4,562 (31.8%)	4,603 (32.1%)	0.01	0.604
Depression	5,352 (26.2%)	4,857 (11.3%)	0.39	<0.001	2,855 (19.9%)	2,927 (20.4%)	0.01	0.289

†Multisource Comorbidity Score is a comorbidity index obtained from inpatient diagnostic information and outpatient drug prescriptions, and validated using Italian data. Patients were categorized as having good (MCS: 0-4), intermediate (5-9), poor (10-14) or very poor (≥ 15) clinical profile. ‡ Comorbidity and Multisource Comorbidity Score both measured according to hospital admission and drug prescriptions experienced five years before the date of index admission; * According to the chi-square test or its version for the trend.

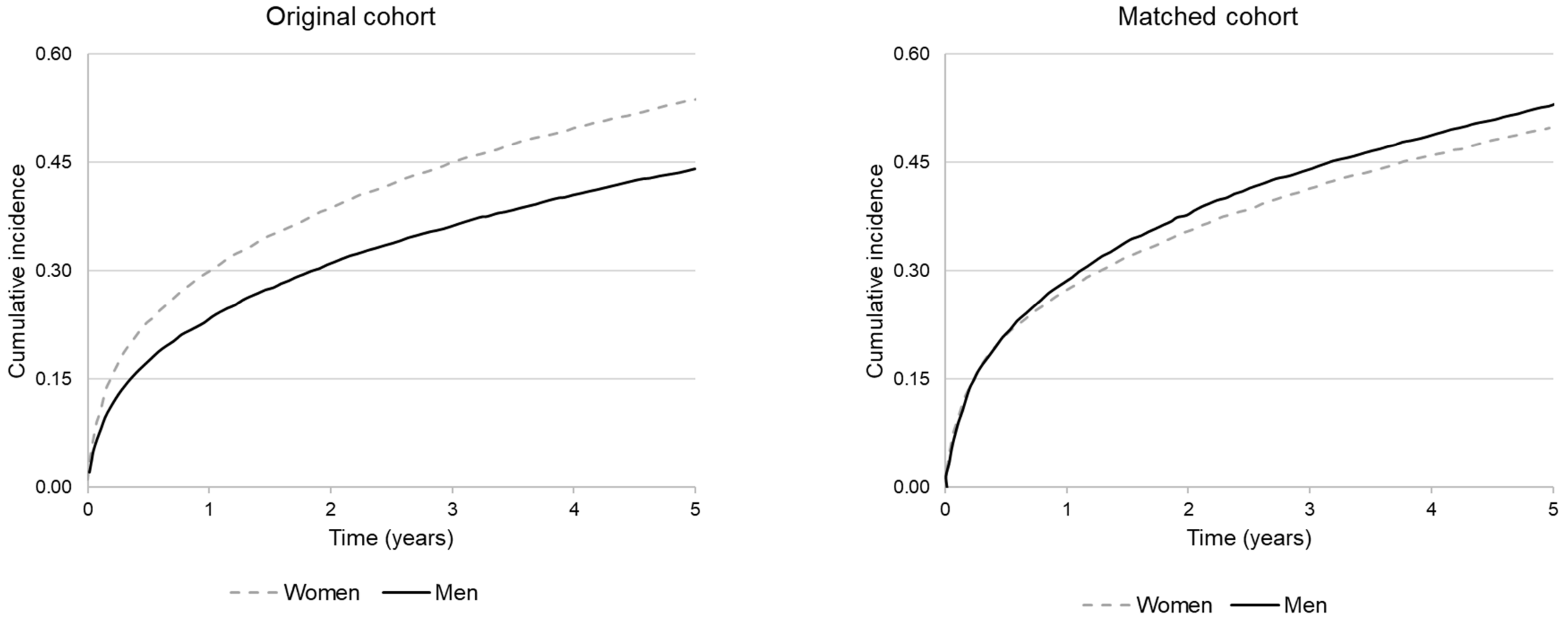

Figure S1. Cumulative incidences of health-related outcomes among Propensity Score 1:1 matched and non-matched cohorts, according to sex.
